# Disorder Analysis in Infrared Spectroscopy of Acetylene Ice

**DOI:** 10.1021/acs.jpca.4c02709

**Published:** 2024-08-16

**Authors:** S. L. A. Mello, R. C. Pereira, C. F. S. Codeço, R. Martinez, E. F. da Silveira, M. M. Sant’Anna

**Affiliations:** † Departamento de Física, Universidade Federal de Viçosa, 36570-900 Viçosa, MG, Brazil; ‡ Departmento de Física, Pontifícia Universidade Católica do Rio de Janeiro, 22451-900 Rio de Janeiro, RJ, Brazil; § Instituto de Física, Universidade Federal do Rio de Janeiro, 21941-972 Rio de Janeiro, RJ, Brazil; ∥ Departamento de Física, Universidade Federal do Amapá, 68903-419 Macapá, AP, Brazil

## Abstract

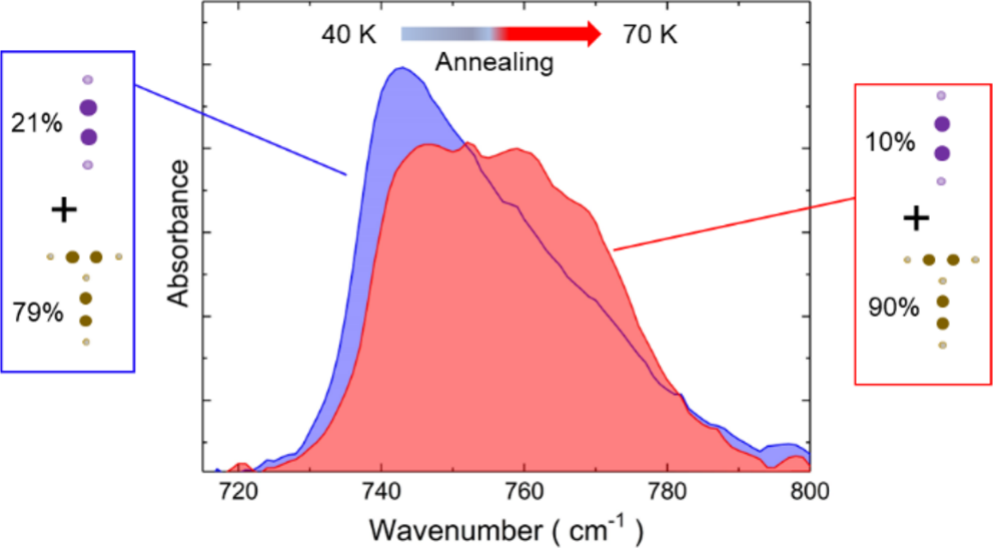

A new method to investigate disorder in ice films is proposed and applied to acetylene ice. It is based on a quantitative analysis of the infrared spectrum data, which includes: the Brendel–Bormann model for the material’s dielectric function; molecular vibration modes calculated by density functional theory (DFT); a monomer–dimer model for amorphous ice; and a peak-shape analysis through Levenberg–Marquardt nonlinear regression. Acetylene ice films with different degrees of disorder were investigated with the proposed method. The results provide an estimate of the degree of disorder in the films and indicate the possibility of existence of a second amorphous phase of acetylene ice grown at temperatures of about 15 K and then annealed. This phase would be similar to the high-density amorphous phase observed for water ice. The infrared data in this work is compared with those from the literature for acetylene gas, acetylene film, and acetylene aerosol. A qualitative analysis reveals differences in the degree of disorder in each system and points to a crystallinity limit for acetylene ice film; that is, the crystalline acetylene film has a higher degree of intrinsic disorder than the crystalline acetylene aerosol.

## 1 Introduction

Infrared spectroscopy shows different signatures for the same material in ordered and disordered configurations. Spectra for monocrystalline samples show sharper features with a frequency profile characteristic of long-lifetime resonances, while highly disordered configurations show broader and asymmetric peaks with a profile approximately Gaussian.^1,2^ Disorder breaks atomic translational symmetry and may also allow the appearance of peaks that are otherwise forbidden by selection rules. Frequency displacement of peaks is often observed as a function of disorder.^1^ Disorder analysis of solids may be crucial in the interpretation of infrared spectra for which atomic defects are present, especially if the degree of disorder in a studied sample is unknown. For example, Poduska et al.^3^ showed that ratios of peak widths and heights corresponding to different vibrational modes in infrared spectra can be used to differentiate calcites with different origins, correlating these origins to different degrees of disorder. Their simple infrared-based diagnostic tool has been applied successfully in geology, and in materials/biomaterials science.^4^ However, analysis of only peak heights and widths, neglecting particularities of profile shapes, is not sufficient to fully characterize the infrared spectrum changes induced by physical processes, such as ion irradiation,^5−7^ growth temperature,^8,9^ etc. In these cases, the shape of a given peak changes as a function of a particular physical parameter (e.g., irradiation dose), maintaining features of both ordered (crystalline) and disordered (polycrystalline or amorphous) configurations.

Defects in the lattice of a highly crystalline sample may generate features in its infrared spectrum; however, generally the degree of disorder in this case is so small that it is neglected in the analysis. In this work we are interested in the intermediary case, in which the infrared spectrum is not representative of a pure crystalline sample, and, for this very reason, there is no established analysis procedure to quantify the degree of disorder in the sample. Here it is worth discussing the meaning of disorder. For a molecular crystal, it means a deviation in the arrangement of molecules from the perfectly ordered state. Such a deviation can be in position (molecules occupying off-site positions) or in direction (angular misalignment of molecules).

An interesting situation occurs with the aerosols observed in Uranus (particles of 0.1 μm diameter) and Neptune (0.4 μm) that have been identified as small C_2_H_2_ particles.^10^ Indeed, the analysis of the infrared radiation from aerosols in space is a powerful technique both to identifying species and to imaging applications, such as in recent observations using the James Webb Space Telescope (JWST), with the Webb’s NIRCam (Near-Infrared Camera).^11,12^

Recently, near-infrared spectra obtained with the JWST NIRSpec (Near-Infrared Spectrograph) revealed the existence of C_2_H_2_ ice in the dwarf planet Sedna.^13^ The authors interpret this observation as the production of acetylene due to the irradiation of methane by energetic charged particles. However, they point out that their spectral feature identification is the result of a first-step analysis and additional insight is dependent on future spectral modeling of their data. We note that disorder changes are inherent to molecular products created through ion irradiation. The method for disorder analysis proposed in the present work contributes to the identification and interpretation of C_2_H_2_ ice infrared spectra with different degrees of disorder. In addition, the proposed method is quite general, not limited to the C_2_H_2_ test case.

Motivated by these findings, we investigate the effect of disorder on the C_2_H_2_ infrared spectrum. A set of C_2_H_2_ ice samples were grown at low temperatures, in amorphous phase, and then annealed to decrease disorder in the sample. Their infrared spectra were measured in situ for different temperatures and the results were compared to C_2_H_2_ spectra from other studies in the literature. An approach combining density functional theory (DFT) and peak-shape analysis shows a highly disordered sample grown at 17 K. After annealing, more vibrational modes become apparent in the spectrum, though it has no resemblance with the spectrum of a crystalline sample. In both situations the ν_5_ absorption band of C_2_H_2_ is consistent with a combination of spectra for the C_2_H_2_ monomer and the (C_2_H_2_)_2_ T-shaped dimer. Our analysis points toward the possible existence of two different solid phases of amorphous acetylene, similar to the low-density amorphous (LDA) and high-density amorphous (HDA) phases observed for water ice.^14^

The paper is organized as follows, in Section 2 we present the experimental and theoretical methods employed in this work. First, the experimental setup to grow C_2_H_2_ ice films is described. Then, the density functional theory calculations are discussed. Last, we present the Brendel-Bormann model used in the peak-shape analysis. In Section 3 the current infrared data for acetylene ice films are presented and analyzed by the method proposed for disorder analysis. The results obtained are discussed in Section 4, along with results from the literature for other forms of acetylene. Finally, the method described in this work to characterize disorder from infrared spectroscopy is summarized in Section 5 and a perspective for future applications is given.

## 2 Experimental and Theoretical Methods

### 2.1 Experimental Setup

Fourier transform infrared spectroscopy (FTIR) measurements were carried out in a UHV chamber at Van de Graaff Laboratory, Pontifical Catholic University of Rio de Janeiro. A KBr disk, 13 mm diameter and 2.0 mm thick, was placed in the center of the UHV chamber and cooled down by a JANIS Closed Cycle Refrigerator Helium Cryostat. The sample temperature variation was performed by a LAKE SHORE Controller model 340. The solid samples were prepared by gas-phase deposition: the residual pressure of the chamber being 10^–6^ mbar, acetylene—with purity higher than 99.7%, purchased from Linde—was blowed onto the KBr with 4.6 × 10^–3^ μm/s rate, for 90 s, aiming to produce films 0.4 μm thick. The thickness of the deposited film was later monitored by infrared spectroscopy (JASCO 4200 FTIR spectrometer) using the ν_3_ or ν_5_ band. The mass density of the C_2_H_2_ ice was considered to be ρ = 0.76 g cm^–3^.^15^

### 2.2 Density Functional Theory Calculations

Eigenfrequencies and corresponding infrared intensities were calculated for C_2_H_2_ vibrational modes of the monomer, the molecular dimer, and the orthorhombic crystal, using density functional theory (DFT) as implemented in the Crystal23 program.^16^ We have used the Crystal23 full geometry optimization (FULLOPTG). Calculations were performed with the combined use of the B3LYP hybrid functional and the triple ζ basis set pob-TZVP-rev2.^17^ For both geometrical optimization and frequency calculations, a value of 10^–11^ Hartree was set to self-consistent-field (SCF) energy-convergence threshold. The truncation of Coulomb and exchange integrals is controlled in the Crystal23 program by the five thresholds parameters Ti (threshold = 10^–Ti^), which were set to 12 (T1–T4) and 20 (T5). For the orthorhombic crystal, the reciprocal space is sampled in a Monkhorst–Pack mesh with shrinking factor equal to 8. The infrared intensities are computed through a coupled-perturbed Hartree–Fock/Kohn–Sham (CPHF/KS) approach.

The coupled perturbed Hartree–Fock method allows us to compute linear and nonlinear optical properties of solid-state systems. Correlation effects have also been included with the extension to CPKS,^18^ i.e., to the density functional theory (DFT) and to hybrid functionals like B3LYP and PBE0. One alternative for the calculation of the IR intensity of crystalline systems implemented in the CRYSTAL code is precisely the CPFH/KS approach, that uses this procedure to compute the dipole moment. Dovesi et al.^19^ compared three methods for the calculation of the IR intensity of crystalline systems. They have shown that at standard computational conditions the three schemes produce IR intensities that differ by less than 1%.

### 2.3 Brendel–Bormann Model

A molecular crystal can be modeled as a system of independent electric-dipole oscillators, under the influence of an alternating electric field. This approach is known as the Lorentz-oscillator model. By solving the general equation of motion for a damping harmonic oscillator, one can write the dielectric function of the system as^20^
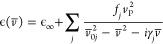
1where ν̅_0*j*_, γ_*j*_, and *f*_*j*_ are, respectively, the resonance wavenumber, the damping constant, and the oscillator strength of a particular vibrational mode *j*, ν_P_ is the plasma frequency, ε_∞_ is the high-frequency dielectric constant, and .

Near each resonance, the imaginary part of eq 1 can be approximated by a Lorentzian function, which is usually used to fit the resonance peaks of Raman and infrared spectra. Although satisfactory results are obtained for highly crystalline solids, the approximation is not suitable for amorphous materials or crystals with some degree of disorder,^1,2^ where the peaks are broader and have tails that decrease slower with the wavenumber. In these cases, a Gaussian function fits better the spectrum peaks than a Lorentzian. If a quantitative analysis of the degree of disorder is intended, both fitting functions are not appropriate. A more suitable approach in these cases is to consider disorder in the Lorentz-oscillator model and extract the crystalline and noncrystalline contributions from the analysis.

In the current study, the Brendel–Bormann model has been used because it is an extension of the Lorentz-oscillator model that includes disorder. Physically, the disorder disturbs locally the dipoles, modifying their vibrational modes and causing shifts in the resonance wavenumbers. Due to the randomness of the disorder, a certain shift may occur toward higher or lower wavenumbers, which results in the broadening of the resonance peak. This effect is treated in the model by considering a Gaussian distribution of frequencies around the resonance wavenumber. Thus, the dielectric function is given by the convolution of eq 1 with a Gaussian distribution, for each resonance
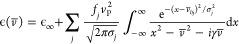
2where σ_*j*_ is the Gaussian standard deviation for the resonance.^21−23^

## 3 Results

### 3.1 Infrared Absorption Spectrum of Amorphous C_2_H_2_ Ice Film

**Figure 1:**
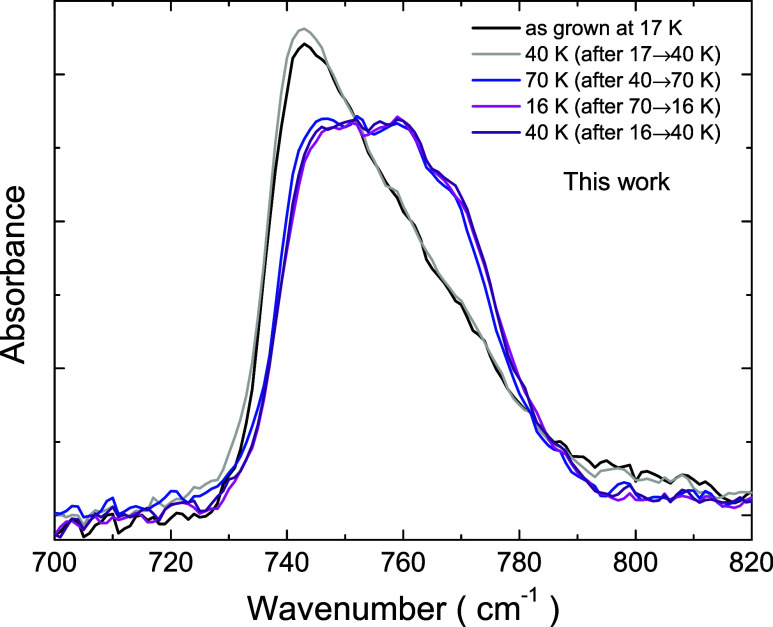
Infrared absorbance spectra of a C_2_H_2_ ice film recorded during a temperature cycle. The sample was grown at 17 K, warmed up to 70 K, cooled down to 16 K, and then warmed up again.

Different from a crystalline sample, which presents sharp peaks in its absorbance spectrum, an amorphous solid presents spectral bands that are broad and asymmetrical.^8,9,24,25^ Typical spectra for a thin C_2_H_2_ ice film are shown in Figure 1, for the ν_5_ band. The spectral resolution is 1 cm^–1^. The film was grown at 17 K, and then annealed up to a maximum temperature of 70 K. The set of spectra corresponds to a temperature cycle, with each spectrum measured after 5 min at the set point to reach temperature stabilization. After reaching 70 K there is an irreversible change of the band shape. Although the band becomes broader, it shows signatures of vibration modes that were not apparent. Increasing the annealing time does not improve the features in the spectrum. The same goes for the annealing temperature, whose increase leads to sublimation of the deposited C_2_H_2_. It means that the spectrum change is robust, suggesting a permanent reconfiguration of the molecules in the solid like in a phase transition. By analyzing the spectral data only, it does not resemble an amorphous-to-crystalline phase transition. An explanation, proposed by Hudson et al.,^8^ assumes that the annealed film is partially crystalline; that is, in the process of crystallization some amorphous fraction remains. Alternatively, we propose that the annealed ice film undergoes a phase transition to a second amorphous phase. The results from disorder analysis obtained in both the current work and in that of Hudson et al. point to the latter explanation.

### 3.2 DFT Calculations for Acetylene Monomer, Dimer, and Crystal

**Figure 2:**
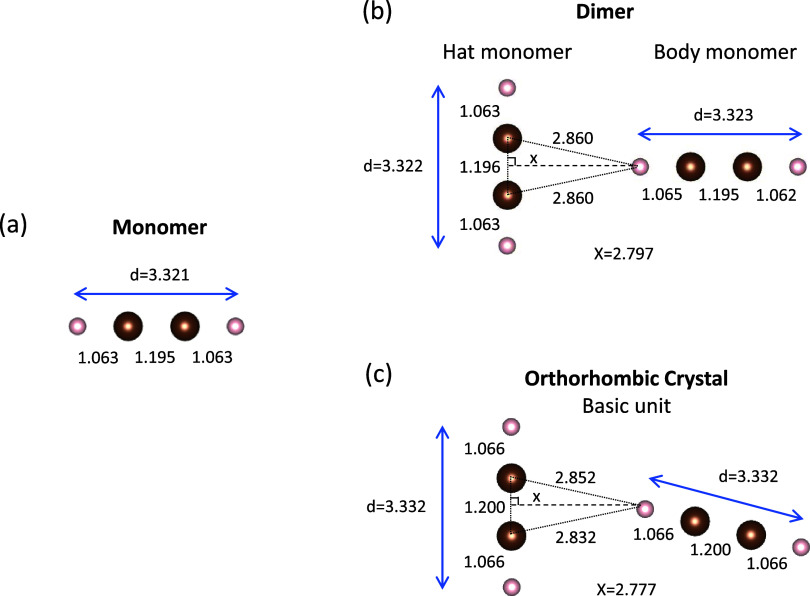
Geometry optimized by DFT calculations for (a) acetylene monomer, (b) dimer, and (c) the basic unit of the primitive cell of a cmce orthorhombic crystal.

Figure 2 shows DFT geometry results (B3LYP functional) for acetylene monomer (Figure 2a), dimer (Figure 2b), and crystal (Figure 2c). The Vesta code is used for visualization.^26^ All distances are given in angstroms. The energy minimum for the (C_2_H_2_)_2_ dimer corresponds to a T-shaped structure with the *C*_2*v*_ symmetry. The intermolecule interaction has been previously highlighted by denominating this configuration as a π-type hydrogen-bonded arrangement with *C*_2*v*_ symmetry.^27^ There is an asymmetry between the two monomers forming the dimer. They are labeled in Figure 2b as body and hat monomers. For the body monomer, the C–H distance is different for the two hydrogens (1.065 and 1.062 Å). This induced asymmetry results in a small static dipole moment. For the (C_2_H_2_)_2_ hat monomer, on the other hand, the C–H distance (1.063 Å) is degenerate, just like for the C_2_H_2_ monomer (Figure 2a). Previous X-ray and neutron diffraction measurements show that the most stable phase for acetylene below 133 K is an orthorhombic crystal with *Cmce* symmetry.^15,28^ Our DFT calculations provide crystallographic cell lattice parameters *a* = 5.66984478 Å, *b* = 6.13022152 Å, and *c* = 6.36294439 Å. Figure 2c shows the basic unit of the optimized geometry for the optimized primitive cell. There is some similarity with the T-shaped dimer. However, the two monomers in the cell form a tilted T-shape. Note that the infinite nature of the crystal (periodic boundary conditions in calculations) leads to the symmetry between the two monomers in the primitive cell, both presenting the same C–H and C–C equilibrium distances (see Figure 2c).

Table 1 shows DFT infrared absorption results for acetylene monomer, dimer, and orthorhombic crystal, calculated using the B3LYP functional. Results are grouped by characteristic bands ν_1_–ν_5_. In this work, our analysis is focused on the bending mode ν_5_. Although other bands can be analyzed, only the ν_5_ band has an appreciable splitting for all acetylene configurations, which allows one to seek for infrared spectrum features induced by structural changes in the solid. For the monomer, the ν_5_ in-plane and out-of-plane vibrational modes are degenerated. For the dimer, this degeneracy is lifted, and combinations of oscillations of the body and hat monomers result in four infrared-active modes.

**Table 1:** Infrared Vibration Modes Calculated by DFT, with B3LYP Functional, for Acetylene Monomer, Dimer, and Orthorhombic Crystal^a^

C_2_H_2_	monomer		dimer		orthorhombic crystal	
band	ν̅ (cm^–1^)	IR intensity (km/mol)	ν̅ (cm^–1^)	IR intensity (km/mol)	ν̅ (cm^–1^)	IR intensity (km/mol)
ν_4_	630.5	0	634.1	0	675.4	0
	630.5	0	638.2	0.33	684.3	0
			643.3	3.28	709.8	0
			651.1	5.16	725.4	0
ν_5_	764.6	88.14	764.4	43.38	780.4	306.48
	764.6	88.14	772.0	103.95	801.6	216.66
			783.4	120.46	810.2	0
			793.3	65.02	814.1	85.06
ν_2_	2092.2	0	2085.7	4.43	2054.3	0
			2088.3	0.19	2064.9	0
ν_3_	3429.9	81.50	3406.2	166.68	3377.4	341.00
			3426.0	90.31	3378.2	395.71
ν_1_	3537.5	0	3522.7	1.37	3489.7	0
			3533.4	0.01	3495.6	0

a Eigenfrequencies are given in units of cm^–1^ and absorption intensities in km/mol.

**Figure 3:**
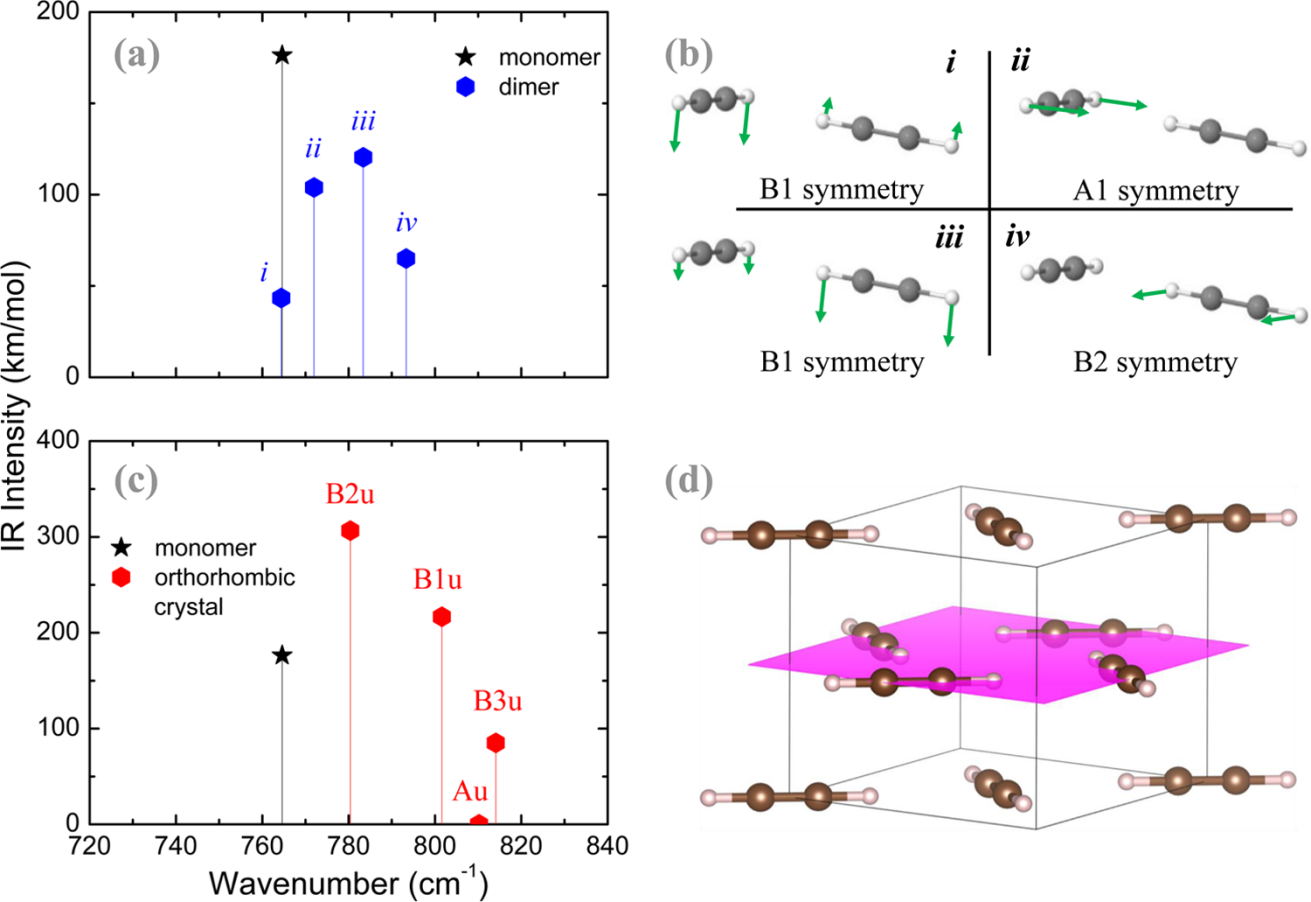
Results of B3LYP DFT calculations for (a) acetylene monomer and dimer, and for (c) acetylene orthorhombic crystal. Representation of (b) the four vibration modes of the acetylene dimer and (d) the structure of the acetylene orthorhombic crystal.

The calculated infrared intensities are plotted as a function of the eigenfrequencies for monomer and dimer (Figure 3a) and for monomer and the orthorhombic crystal (Figure 3c). Figure 3b shows geometric characteristics of the four dimer ν_5_ modes. An animation of these vibrational modes is presented in the Supporting Information. The symmetry of each calculated mode is shown in the figure near to the corresponding peak. A label from i to iv is included to indicate the most similar oscillating mode of the dimer. Due to crystalline symmetry, one of the four crystalline ν_5_ modes is infrared inactive: Au symmetry, out-of-plane antisymmetric bending mode. Figure 3d shows geometric characteristics of the orthorhombic C_2_H_2_ crystal.

### 3.3 D3 Dispersion Corrections for DFT Calculations

The Crystal23 code allows the inclusion of long-range electron correlation effects that are missing in DFT methods. The so-called D3 semiclassical correction introduces in the calculations the effects of the weak London forces.^29,30^ The D3 corrections are numerically small for both frequencies and intensities calculated for the C_2_H_2_ dimer in this work. However, they lead to a qualitative change in the equilibrium configuration. There is a change in symmetry group of the most stable configuration from *C*_2*v*_, T-shaped, to Cs, (slightly) tilted T-shaped. We discuss this change below. For the C_2_H_2_ orthorhombic crystal calculations, there are also no appreciable changes in intensities. For the frequencies, however, there are blue-shifts for all peaks of the ν_5_ band. These shifts are also discussed below.

The introduction of the D3 correction in DFT calculation, assuming *C*_2*v*_ symmetry (T-shaped) for the C_2_H_2_ dimer, results in a negative-frequency normal mode. This is a well-known indicator that the symmetry assumed in the calculations is not appropriate to describe the system.^31^ In this case, a standard scan procedure implemented in Crystal23 is to perform multiple calculations of the total energy slightly changing the atom positions of the system and searching for minima in the energy curve.^31^ Once minima are identified in the plot, the output of the program for an energy minimum is analyzed and the symmetry of the atomic configuration identified. This new symmetry group can be used as a starting point for a new calculation of the frequency spectrum. If the negative frequency vanishes, the new group symmetry is identified.

**Figure 4:**
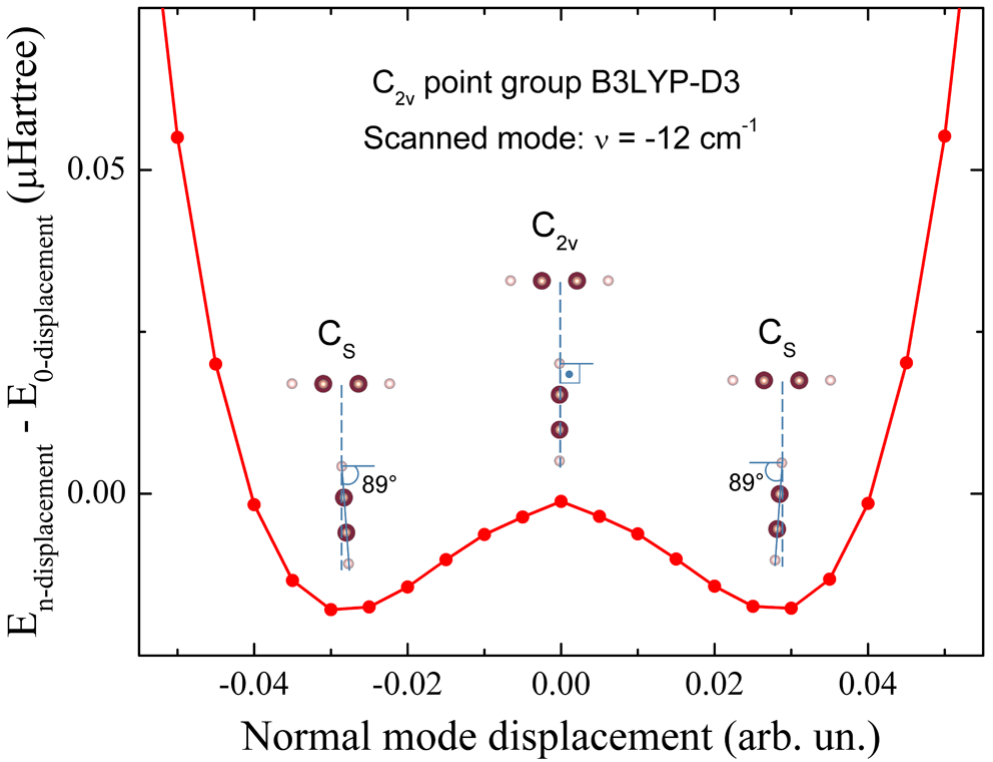
Crystal23 scan-mode test for the vibrational mode with the lowest frequency when *C*_2*v*_ symmetry (T-shaped) is assumed for the C_2_H_2_ dimer. The two minima indicate a lower symmetry when D3 correction is included. B3LYP-D3 calculations assuming *C_S_* symmetry eliminate the spurious ν̅ = −12 cm^–1^ obtained when assuming *C*_2*v*_ symmetry. The energy change is plotted as a function of a geometry change (arbitrary units) for the vibration mode.^31^

The Crystal23 scan procedure is used for the ν̅ = −12 cm^–1^ mode, calculated assuming *C*_2*v*_ symmetry. The results are shown in Figure 4. The *C*_2*v*_ leads to a maximum, not a minimum. Two shallow minima are observed, which correspond to (degenerated) tilted-T configurations. Calculations using the D3 correction and assuming the *C_S_* symmetry group, describing the tilted-T configuration, are also performed and the results are presented in Table 2. However, the shallow minima in Figure 4 correspond to a tilt between C–C directions of the two C_2_H_2_ molecules very close to 90°, tilted by only 1°. This tilt is not noticeable in the scale of Figure 2b. Previous theoretical works, without using long-range corrections, have obtained the T configuration for acetylene dimer, but the tilted-T configuration was only obtained for the dimer of diacetylene, a longer linear hydrocarbon molecule.^27^

**Table 2:** Infrared Vibration Modes Calculated by DFT, with B3LYP Functional and D3 Correction, for Acetylene Monomer, Dimer, and Orthorhombic Crystal^a^

C_2_H_2_	monomer		dimer		orthorhombic crystal	
band	ν̅ (cm^–1^)	IR intensity (km/mol)	ν̅ (cm^–1^)	IR intensity (km/mol)	ν̅ (cm^–1^)	IR intensity (km/mol)
ν_4_	630.2	0	634.3	0	690.4	0
	630.2	0	639.1	0.64	691.9	0
			640.3	3.11	732.3	0
			651.2	5.68	740.2	0
ν_5_	764.3	88.14	764.1	38.92	800.7	332.27
	764.3	88.14	772.2	103.88	802.7	255.89
			782.6	124.91	822.2	75.72
			796.2	62.22	833.7	0
ν_2_	2092.7	0	2085.7	6.01	2050.5	0
			2088.5	0.01	2063.0	0
ν_3_	3430.7	81.49	3402.8	196.72	3371.4	481.12
			3426.8	91.56	3376.2	562.92
ν_1_	3538.2	0	3521.7	2.58	3489.0	0
			3534.1	0.01	3495.7	0

a Eigenfrequencies are given in units of cm^–1^ and absorption intensities in km/mol.

**Figure 5:**
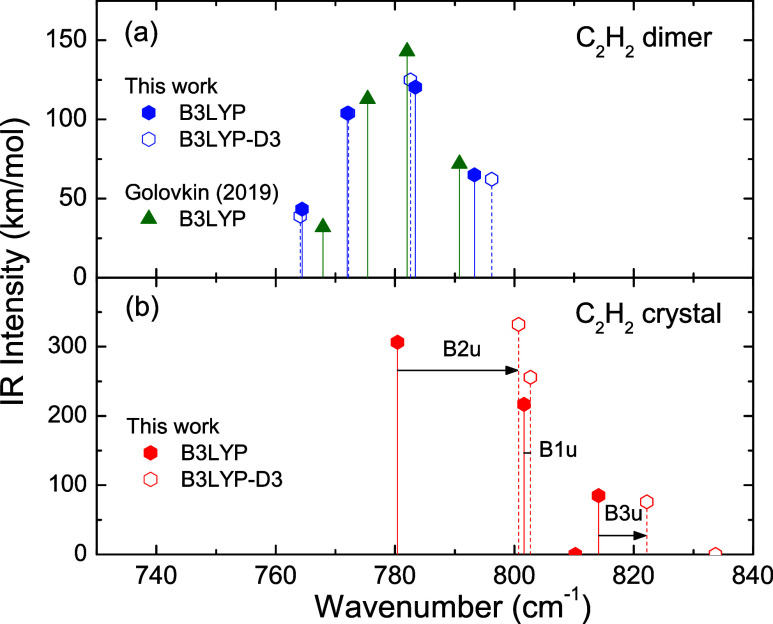
Results of B3LYP DFT calculations with (open symbols) and without (filled symbols) D3 corrections: (a) C_2_H_2_ dimer and (b) C_2_H_2_ orthorhombic crystal. Results from Golovkin et al.,^32^ for C_2_H_2_ dimer, using Gaussian 09 program are presented in (a) for comparison.

The effects of the D3 correction on C_2_H_2_ dimer frequency and intensity values (which are also small) are presented in Figure 5(a). The DFT predictions of Golovkin et al.^32^ are also presented. They are based on the B3LYP functional using a cc-pVTZ basis, implemented with the Gaussian 09 program. The three sets of results, our calculations with and without D3 correction and the data of Golovkin et al. (without D3 correction), show similar frequencies and intensities. The difference between our calculations with and without D3 correction is smaller than the difference between our calculations and those performed by Golovkin et al. with a slightly different basis. Thus, for the analysis of experimental spectra in the following sections we use our DFT calculations in the simpler version without the D3 correction. Nevertheless, we point that, in an actual amorphous configuration formed by C_2_H_2_ molecules, a T-shaped dimer configuration eventually connecting two molecules may be affected by the surrounding medium, eventually with more pronounced deformation toward the tilted *C_S_* symmetry.

The effects of the D3 correction on C_2_H_2_ orthorhombic crystal frequency and intensity values are presented in Figure 5(b). Changes in intensities are not appreciable. However, the D3 correction results in blue-shifts for all symmetries of vibrational modes: B1u, + 1.0 cm^–1^; B2u, + 20.3 cm^–1^; B3u, + 8.1 cm^–1^; Au (infrared inactive mode), +23.5 cm^–1^. Thus, Figure 5 shows that the inclusion of D3 corrections increases the displacement of ν_5_ frequencies for the C_2_H_2_ orthorhombic crystal regarding the C_2_H_2_ dimer values.

### 3.4 Monomer–Dimer Model for Amorphous Acetylene Ice

Amorphous acetylene ice is obtained when the film is grown at low temperatures (below 40 K). Its spectrum has a structureless peak with a long tail toward higher wavenumbers. If the film is annealed, the band shape changes as represented in Figure 1. Even for spectra like those, with no resolved peaks, there should be certain vibration modes of acetylene structures which are dominant in the absorption of light. A simple model is to consider amorphous ice as an ensemble of C_2_H_2_ monomers and (C_2_H_2_)_2_ dimers, which are the simplest structures for acetylene clusters; a similar simplification has been used to describe liquid water from water clusters, with eventual dimer formation led by hydrogen bond.^33,34^ A combination of acetylene monomer and dimer vibration modes results in the measured spectrum. Although other structures like trimers and tetramers may occur,^32^ they can be neglected in the model. Furthermore, the structures forming the primitive cell of acetylene crystal resemble a T-shaped dimer (Figure 2).

**Figure 6:**
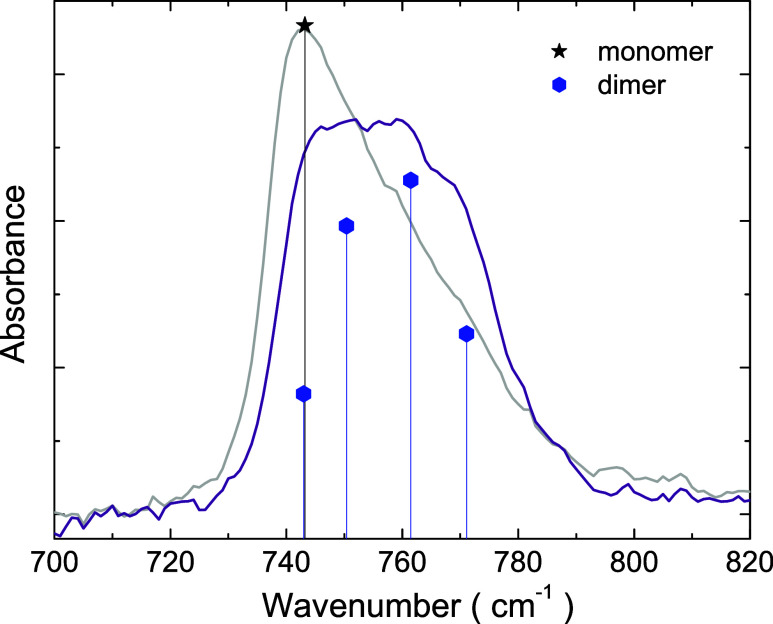
Comparison of DFT calculations for acetylene monomer and dimer (results for the ν_5_ band presented in Table 1) with the measured infrared spectra of acetylene ice (spectra shown in Figure 1 for 40 K). For the sake of comparison, it was used a scaling factor of 0.972 for both the monomer and dimer eigenfrequencies calculated by DFT; in addition, all IR intensities were normalized by the monomer IR intensity, and the experimental data were normalized by the intensity of the absorbance peak for the as grown sample.

Figure 6 presents the calculated vibrational modes of the ν_5_ band for two acetylene structures: Monomer and dimer. At the equilibrium configuration, the four atoms of C_2_H_2_ are colinear forming the monomer, while the dimer is composed of two monomers in a T-shape. For comparison with the measured acetylene spectrum shown in Figure 6 (the same as those presented in Figure 1), the calculated IR intensity is plotted as a function of the scaled eigenfrequency; that is, the eigenfrequencies were multiplied by a common factor of 0.972 (see ref (32) for a discussion of the scaling procedure). It is noticeable that there is a correspondence of the spectrum of the as-grown sample with the monomer, and the spectrum of the annealed sample with the dimer. This is evidenced by the close matching of the peak positions in the spectra with the calculated DFT eigenfrequencies. For the annealed sample, one observes that the relative IR intensities match well the results for dimer, with the middle peaks being more intense than the lateral ones.

A comparison between the spectrum of the as-grown film and DFT calculations suggests that the monomer is the dominant structure for the spectrum, but the dimer is also present and explains the peak tail. After annealing the sample, the absorbance signal decreases in the monomer position and increases in the range of dimer eigenfrequencies. This is explained by the formation of dimers from pairs of monomers as a consequence of annealing. Changes in the fraction of monomers and dimers result in changes in the band shape.

### 3.5 Semi-Empirical Approach for Disorder Analysis

The disorder analysis consists in fitting the absorbance spectrum by considering the acetylene ice composed of only monomers and dimers with unknown fractions, *w*_m_ and *w*_d_ = 1 – *w*_m_, respectively. The measured absorbance spectrum is then a linear combination α_meas_ = *w*_m_α_m_ + *w*_d_α_d_, where α_m_ and α_d_ are, respectively, the monomer and dimer contributions to the absorbance signal. It is used the classical expression of the absorption coefficient, α(ν̅) = 4πv̅*k*(v̅), where *k* is the imaginary part of the complex refractive index, *Ñ* = *n* – *ik*. The dielectric function, in turn, is related to the complex refractive index by *Ñ* = √ϵ, and is calculated by eq 2. A script in GNU Octave language was written to perform the fits, through Levenberg–Marquardt nonlinear regression. To optimize the computation, the analytical solution of the integral in eq 2 was used, as described in refs (21−23).

The model parameters are *w*_m_, *w*_d_, *f*_*j*_, ν̅_*j*_, γ_*j*_, σ_*j*_, whose definitions are given in the text. As discussed previously, the acetylene monomer has one vibration mode in the ν_5_ band, while the dimer has four, which results in 22 parameters to fit the absorbance data. The values of infrared intensity and eigenfrequency calculated by DFT for acetylene monomer and dimer provide us the parameters *f*_*j*_ and ν̅_*j*_; thus, the number of unknown parameters is reduced to 12. As discussed by Brendel and Bormann,^21^ and verified in this work, their model gives good fits for parameters γ_*j*_ fixed. Here we used γ_*j*_ values about 3 cm^–1^ for all modes, which is approximately the width of the absorbance peaks for C_2_H_2_ crystalline samples. Recalling that *w*_d_ is fixed by definition, *w*_d_ = 1 – *w*_m_, then there are only six free parameters left in the model: *w*_m_ and σ_*j*_ (for one monomer mode plus four dimer modes). Three additional parameters are included to scaling DFT eigenfrequencies and the measured absorbance spectrum, so that ν̅_*j*_ → *c*_0_ν̅_*j*_ and α → *c*_1_α + *c*_2_.

**Figure 7:**
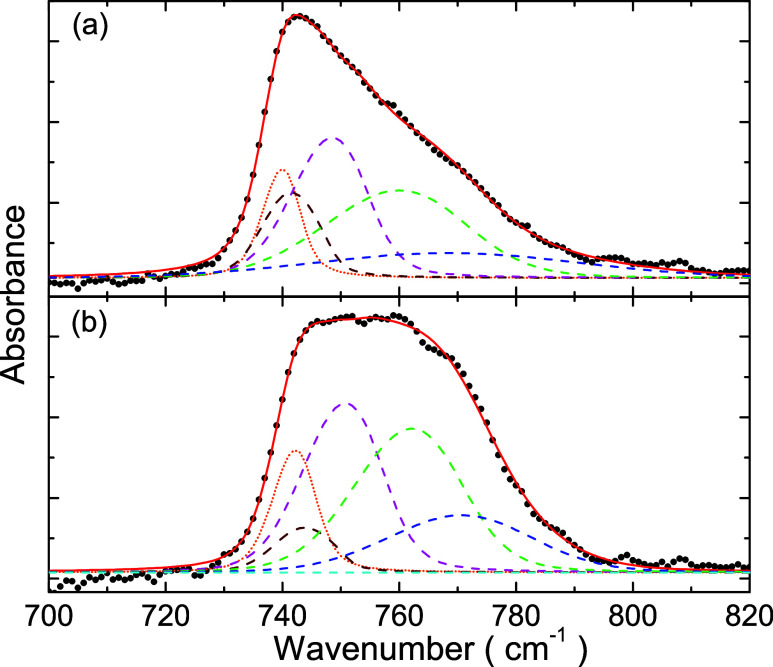
Nonlinear fits (solid line) to the infrared spectrum data (symbols) of an acetylene ice film (a) before and (b) after annealing. The data correspond to those presented in Figure 1 for 40 K. The dotted and dashed lines correspond to the vibration modes for the monomer and dimer, respectively.

Figure 7 shows fits to the absorbance spectra of C_2_H_2_ thin films by using the procedure discussed above. The data correspond to those for 40 K presented in Figure 1, before (Figure 7a) and after (Figure 7b) annealing at 70 K. The solid line over symbols (experimental data) is the nonlinear fit, the dotted line is the contribution to the absorbance spectrum due to monomers, and the dashed lines correspond to the four vibration modes from dimers. The fitting parameters are listed in Table 3. The parameters presented with their corresponding errors are the free parameters of the fits. The proposed model provides good agreement with the experimental data, as shown in Figure 7 for the annealed and nonannealed films. The degree of disorder can be quantified by the fraction of monomers *w*_m_ and the Gaussian standard deviation σ_*j*_ from the Brendel–Bormann model; the smaller are these parameters, the lesser the disorder. After annealing the band broadens on the top, owing to the increased contribution of dimer vibration modes. This is verified by a decrease in *w*_m_ from 21 to 10% (and the corresponding increase in the fraction of dimers), and a decrease in σ_3_ and σ_4_. The other Gaussian standard deviations have not changed appreciably.

**Table 3:** Parameters Used to Fit the Acetylene Spectra Presented in Figure 7^a^

		monomer	dimer			
	mode number	1	1	2	3	4
before annealing:	*f*_*j*_	176.28	43.38	103.95	120.46	65.02
*w*_m_ = 0.21 ± 0.01	*c*_0_ν̅_*j*_ (cm^–1^)	739.12	739.22	746.57	757.59	767.17
*c*_0_ = 0.9670 ± 0.0001	γ_*j*_ (cm^–1^)	3.2	3.2	3.2	3.2	3.2
*c*_1_ = (1246 ± 9) × 10^–7^	σ_*j*_ (cm^–1^)	2.3 ± 0.2	3.1 ± 0.3	5.4 ± 0.2	11.2 ± 0.2	22 ± 1
after annealing:	*f*_*j*_	176.28	43.38	103.95	120.46	65.02
*w*_m_ = 0.10 ± 0.02	*c*_0_ν̅_*j*_ (cm^–1^)	741.34	741.43	748.81	759.86	769.47
*c*_0_ = 0.9700 ± 0.0003	γ_*j*_ (cm^–1^)	3.2	3.2	3.2	3.2	3.2
*c*_1_ = (140 ± 2) × 10^–6^	σ_*j*_ (cm^–1^)	2.9 ± 0.3	3 ± 1	5.8 ± 0.2	8.2 ± 0.2	11.6 ± 0.4

a The free parameters in the model are presented with their corresponding errors; the other ones are fixed.

## 4 Discussion

The C_2_H_2_ molecule in the monomer configuration presents two absorption bands active in the infrared, namely, ν_3_ and ν_5_. For the dimer configuration and the orthorhombic crystal of acetylene other bands are present, but the ν_3_ and ν_5_ are the most intense ones (see Table 1). We focus the discussion on the ν_5_ band, which comprises more active infrared vibration modes than the other ones, and it is the most affected by disorder.

**Figure 8:**
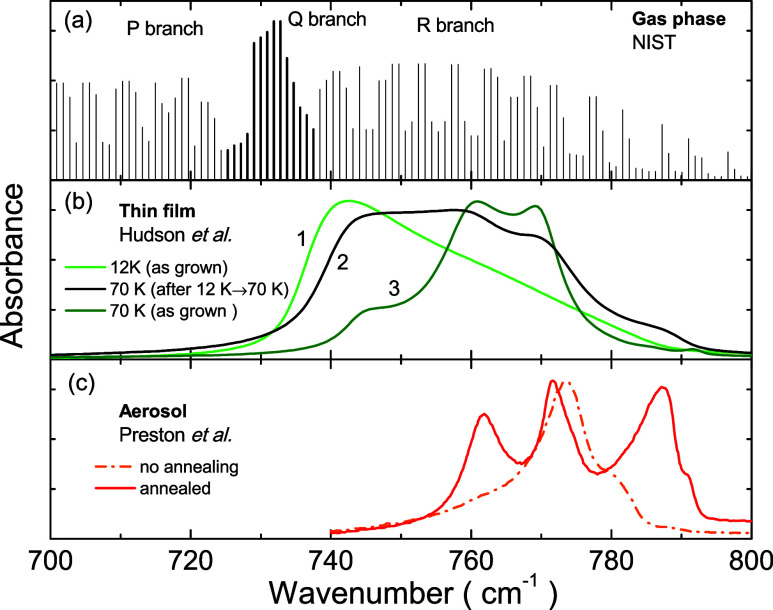
Infrared absorbance spectrum from the literature for (a) acetylene gas, (b) acetylene thin film, and (c) acetylene aerosol. IR spectrum data for (a, b) are available in the NIST and NASA Web sites, respectively,^35,36^ while those of (c) were digitized from ref (37).

Figure 8 shows experimental data from the literature for infrared spectra of acetylene in three different forms: (a) gas, (b) thin film, and (c) aerosol. In the gas phase the molecule can rotate, in addition to the bending vibration. This additional degree of freedom allows it to perform rotational–vibrational transitions between modes that are very close in energy (approximately 1 cm^–1^ in wavenumber), being represented by vertical lines in the spectrum (Figure 8a). The rotational levels are described by the quantum number *J*. By the selection rule, only transitions with Δ*J* = ±1 or 0 (in the case of polyatomic molecules) are allowed. This gives origin to the P (Δ*J* = −1), Q (Δ*J* = 0), and R (Δ*J* = +1) branches in the spectrum. As for the Q branch there is no gain or loss in the rotational energy, thus it corresponds to pure vibration modes that form the ν_5_ band.

From acetylene gas to thin film spectra, the ν_5_ band shifts to higher wavenumbers (blue-shift), as seen by comparing the Q branch position in Figure 8a with the spectral positions in Figure 8b. The same fact occurs from acetylene thin film to aerosol spectra (Figure 8b,8c). According to our DFT calculations and model for the formation of C_2_H_2_ ice, we interpret such shifts as a result of a decrease in disorder. In the gas phase the molecules are distributed randomly, thus the system is fully disordered. The less disordered system, and then the more blue-shifted, is the annealed C_2_H_2_ aerosol, which is made of highly crystalline nanoparticles. In the intermediary case is the thin film.

**Figure 9:**
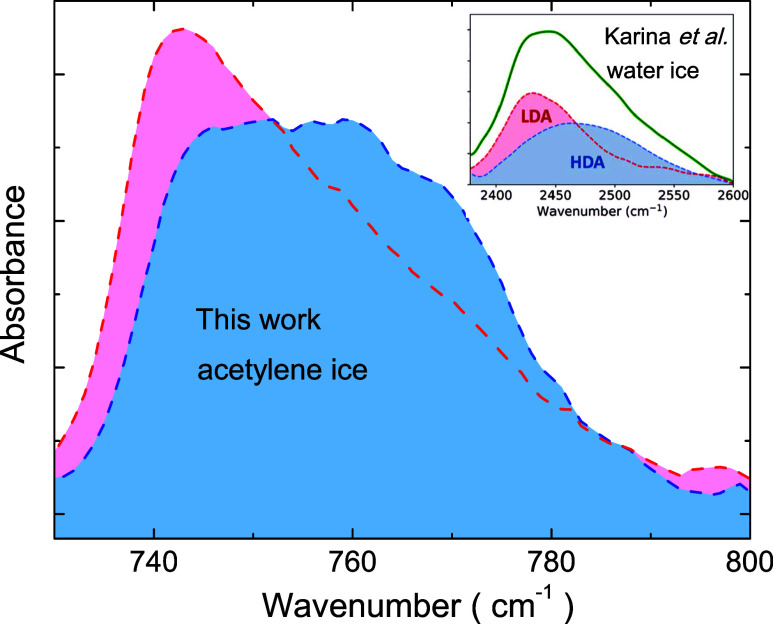
Infrared absorbance spectra for acetylene ice films (same as those of Figure 1 for 40 K), and for deuterated water ice (inset).^14^ Adapted with permission from ref (14). Copyright 2022 American Chemical Society.

A natural parameter to visualize the effect of disorder is the average distance between neighboring C_2_H_2_ molecules. Theoretically, it decreases from ∞ in the gas to a minimum value in the crystal, as the disorder decreases. Although not intended for disorder quantification, this parameter helps us understand the changes in the acetylene absorbance spectra. When C_2_H_2_ molecules are in the gas phase they are isolated from each other, then only monomers contribute to the spectrum. In a solid, however, the average distance between molecules is decreased, which favors the formation of dimers from pairs of monomers. The results presented in this work suggest that both monomers and dimers contribute to the absorption spectrum of C_2_H_2_ thin films, and their fractions indicate the degree of disorder in the system. Hudson et al.^8^ obtained acetylene ice films of different crystalline quality by annealing them or, alternatively, by varying the temperature of the substrate on which the films were grown. In brief, the growth is carried out by deposition of gas-phase C_2_H_2_ onto a KBr substrate, like growths performed in this work. Figure 8b presents the spectra of three of their samples: a film grown at 12 K (curve 1); one grown at 12 K and then annealed at 70 K (curve 2); and another one grown at 70 K (curve 3). The first and the last are referred to as amorphous and crystalline C_2_H_2_ ice, respectively, by Hudson et al. After annealing the amorphous sample, the spectrum changes in the same way as that of our annealed sample (see Figure 1). Although this spectrum is not like the one of crystalline C_2_H_2_ ice, features that are characteristic of both amorphous and crystalline samples are observable. Hudson et al. hypothesize that the annealed sample corresponds to crystalline C_2_H_2_ ice in which a fraction of amorphous material has remained. Otherwise, we propose that the annealed film undergoes a phase transition to a second amorphous phase, as observed for water ice.^14^Figure 9 shows the similarities between the infrared spectra of acetylene ice films presented in this work and those of deuterated water ice from Karina et al.^14^ (inset in Figure 9). The low-density amorphous phase corresponds to C_2_H_2_ ice grown at low temperatures, while the high-density amorphous phase corresponds to the annealed ice. A crystalline phase is only achieved for elevated substrate temperatures (about 70 K) during C_2_H_2_ deposition. The high-density of the second amorphous phase is justified because of the increased fraction of dimers after annealing. Since the average distance between neighboring C_2_H_2_ molecules is decreased for dimers, the result is a more packed structure.

Figure 8c shows results from Preston et al. for the absorbance spectra of C_2_H_2_ ice produced by aerosol expansion.^37,38^ Annealing was carried out by condensation of ethane onto acetylene aerosol particles. Preston et al. used vibrational exciton calculations to interpret their results in terms of an increase in crystallinity. Predictions without annealing were associated with polycrystalline particles and predictions with annealing associated with monocrystalline particles. Among the presented data in Figure 8, the spectrum of the annealed C_2_H_2_ aerosol is the most representative of crystalline ice. There are three distinguished peaks, well separated in position and with pronounced peak-to-valley heights. Here the fitting model for disorder analysis fails to give good results. First, because C_2_H_2_ aerosol particles are expected to be crystalline, from the spectrum features discussed above, thus the DFT calculations for monomer and dimer are not useful in the model. Instead, the results for the orthorhombic crystal should be used. This is reinforced by the equivalence of the number of peaks in the spectrum and the number of vibration modes from DFT calculations, which are three for the orthorhombic crystal. Second, the asymmetry in the spectrum peaks indicates that only vibration modes for the orthorhombic crystal are not sufficient to fit the spectrum. Additional vibration modes from dimer and monomer, for example, might enter in the model as first- and second-order corrections, respectively. Consequently, the number of parameters would increase considerably, compromising the convergence and the reliability of the results.

Interestingly, the band of the polycrystalline aerosol lays on the right of that of the crystalline film. Considering that the band shifts to higher wavenumbers as disorder decreases, then the polycrystalline aerosol is somehow less disordered than the crystalline film. One should recall that the crystalline ordering can be broken by molecules occupying off-site positions or molecules misaligned angularly. In both cases the absorbance spectrum is deformed. The predictions from Preston et al., along with their experimental results, suggest that the annealing of the polycrystalline aerosol corrects the angular misalignment of C_2_H_2_ molecules, making the structure monocrystalline. For acetylene films (Figure 8b), one might hypothesize that even for the sample referred to as crystalline a certain degree of disorder remains, due to angular misalignment of C_2_H_2_ molecules.

Finally, Mejía et al. show that C_2_H_2_ is synthesized after ion irradiation of pure CH_4_ ice films.^39^ In their work, CH_4_ films are irradiated by 6 MeV O^2+^ ion beams with fluences varying in the range (0.01–22) × 10^12^ cm^–2^. Initially, the absorbance of the ν_5_ assignment at 736 cm^–1^ (which is supposed to be the C_2_H_2_ monomer according to our DFT calculations) increases as the ion fluence is increased until approximately 1 × 10^12^ cm^–2^; then it decreases for further increment in the fluence. Other ν_5_ assignments of higher wavenumbers (corresponding to vibration modes of the (C_2_H_2_)_2_ dimer) appear in the absorbance spectrum just before the peak at 736 cm^–1^ starts diminishing, and their intensities increase as the irradiation advances. The shape of the ν_5_ band obtained for the maximum fluence resembles that of the annealed sample presented in this work. The results from Mejía et al. suggest that the ion irradiation leads to dimerization of C_2_H_2_ monomers, with a high-density amorphous phase of acetylene obtained for a fluence of 22 × 10^12^ cm^–2^. Like annealing, the effect of irradiation in their work is decreasing the system disorder, resulting in a blue-shift of the ν_5_ band.

## 5 Conclusions

We presented a method to investigate crystalline disorder from infrared absorbance spectrum. It is applied to acetylene ice films, but the method is quite general and can be promptly used to study other organic ices (e.g., ethane and methane) and inorganic ices (such as water and ammonia), by modifying the proposed monomer–dimer model for amorphous acetylene to an appropriate expansion in terms of molecular clusters for the ice of interest. This approach opens the possibility to investigate ice in the atmospheres of several planets and moons, and even in the outer solar system, based on the measured infrared spectrum data.

Acetylene ice films grown at low temperatures, and then annealed, were investigated. The results show that both annealed and nonannealed films are highly disordered. From a semiempirical approach, we could estimate the degree of disorder in the ice films and explain the effect of annealing, considering that the amorphous acetylene is made up of monomers and dimers in a first approximation. Changes in the monomer and dimer populations in the ice result in band shape modifications of the measured infrared bands, consistent with the proposed model as shown by results of nonlinear regression. Differently from the interpretation existing in the literature that the acetylene amorphous film becomes crystalline after annealing,^8,9^ our results indicate that it possibly undergoes a phase transition to a second amorphous phase, similar to the high-density amorphous phase of water ice.^14^ The crystalline phase is only obtained for acetylene films grown at approximately 70 K, that is, just before C_2_H_2_ sublimation. However, even for those acetylene ice films considered crystalline in the literature, there might be some degree of disorder in their structures. This can be inferred by comparing the infrared spectra of acetylene film and acetylene aerosol. Despite both being crystalline, the spectrum of acetylene aerosol has well-defined peaks, with pronounced valleys between them, different from what is seen for the spectrum of acetylene film. A possible explanation is that the remaining disorder in acetylene film comes from angular misalignment of C_2_H_2_ molecules.

In summary, the proposed method for disorder analysis gives good agreement with the experimental data for acetylene, and yet can be extended for other ices. A potential application of the method is the spectral deconvolution of infrared data; for instance, a mixture of acetylene and propane, which have vibration modes in the wavenumber range of 700–800 cm^–1^. A possible limitation is the increased number of free parameters that would be used in the Levenberg–Marquardt nonlinear regression. However, this might be overcome by using only the dominant vibration mode of each mixture component in a first approximation, and then including other modes after convergence is achieved.

## Supporting Information Available

(C_2_H_2_)_2_ dimer ν_5_ vibration modes animation (MP4)
